# Infantile Spasm: A Review Article

**Published:** 2014

**Authors:** Mohammad Mahdi TAGHDIRI, Hamid NEMATI

**Affiliations:** 1 Pediatric Neurology Research Center, Shahid Beheshti University of Medical Sciences (SBMU), Tehran, Iran; 2 Pediatric Neurology Center of Excellence, Department of Pediatric Neurology, Mofid Children Hospital, Faculty of Medicine, Shahid Beheshti University of Medical Sciences (SBMU), Tehran, Iran

**Keywords:** Infantile spasms, Electroencephalography, Hypsarrhythmia

## Abstract

**Objective:**

Infantile spasm (IS) is a convulsive disease characterized by brief, symmetric axial muscle contraction (neck, trunk, and/or extremities). IS is a type of seizure that was first described by West in 1841, who witnessed the seizure in his own son. West’s syndrome refers to the classic triad of spasms, characteristic EEG, and neurodevelopmental regression. Most cases involve flexors and extensors, but either of the types may be involved independently.

IS, as its name implies, most often occurs during the first year of life with an incidence of approximately 1 per 2000-4000 live births. Most, but not all, patients with this disorder have severe EEG abnormalities; this pattern was originally referred to as hypsarrhythmia by Gibbs and Gibbs. Cases with known etiology or signs of brain damage are considered as symptomatic. The Overall prognosis of the disease is poor. Peak onset age of the epileptic syndrome is 3 to 7 months, which mainly occurs before 2 years of age in 93% of patients. Hypsarrhythmia is the EEG hallmark of IS, which comprised a chaotic, bilaterally asynchronous high-voltage polyspike, and slow wave discharges interspersed with multifocal spikes and slow waves.

Etiological classification is as follows: 1) Symptomatic: with identifiable prenatal, perinatal, and postnatal causes with developmental delay at the presentation time; 2) Cryptogenic: unknown underlying cause, normal development at the onset of spasms, normal neurological exam and neuroimaging, and no abnormality in the metabolic evaluation; 3) Idiopathic: pure functional cerebral dysfunction with complete recovery, no residual dysfunction, normal neuroimaging and normal etiologic evaluation, and normal neurodevelopment.

## Introduction

**Definition and classifications**

Infantile spasms (IS) are a unique form of seizure disorder that their occurrence is almost entirely limited to infancy (the first year of life) and they are refractory to conventional anticonvulsant drugs. IS usually are associated with developmental retardation or deterioration and a characteristic electroencephalographic (EEG) pattern (hypsarrhythmia) that together form a syndrome (1).

IS involves a sudden, generally bilateral and symmetric contractions of muscles of the neck, trunk, and extremities (1). Patients with IS can be classified into focal IS and diffuse groups according to different lateralizing signs, because the classification provides practical information on the long-term outcome and treatment strategy (2). The type of seizure depends on which muscle groups (flexor or extensor) are predominantly affected and on the extent of the contraction. Flexor spasms have long been considered as the most characteristic type of seizure and thus, they have been predominantly featured in naming the syndrome. Flexor spasms (42% of cases) and mixed flexor-extensor spasms (50% of cases) included the most common types (1).

IS most commonly develops between 3 and 8 months of age, with only 8% of cases first being encountered in infants older than 1 year of age. (3). The EEG has the characteristics of hypsarrhythmia, a disorganized interictal pattern that consists of random high-voltage slow waves and spikes. In addition to classic EEG pattern, there are several hypsarrhythmia variants, which have been grouped together and termed as “modified hypsarrhythmia” (4,5).

ISs are classified as cryptogenic or symptomatic. Cryptogenic spasms, a minority of the cases of IS, occur in infants with normal birth and development until the onset of seizure, in whom no clear cause for the convulsions can be identified. A variety of prenatal and perinatal insults are accounted for the majority of cases in the symptomatic group (3,6).

**Etiologic factors**

ISs have multiple causes, and their mechanism is at best, incompletely understood(1). Although the unifying epileptogenic mechanism is unknown, various underlying disorders cause ISs. These disorders are often classified into prenatal, perinatal and postnatal groups. Accounting for over 40 percent of total cases, prenatal etiologies include CNS malformations (focal cortical dysplasia, lissencephaly, holoprosencephaly, hemimegalencephaly, Callosal agenesis/Aicardi syndrome), chromosomal abnormalities (trisomy 21, Miller-Dieker syndrome), single-gene errors, neurocutaneous syndrome (TS, NF1, incontinentia pigmenti), congenital central nervous system infections (TORCH), and rarely, in-born error of metabolism. Perinatal precipitants include hypoxic ischemic encephalopathy and hypoglycemia.

Finally, postnatal factors include intracranial infections, hypoxic-ischemic insults, and brain tumors. Overall, cortical malformations, hypoxic-ischemic, and tuberous sclerosis are the most common known associated disorders (4).

**Diagnostic evaluations**

In a child with suspected IS, an EEG is needed to confirm the presence of hypsarrhythmia. If EEG remains normal with no features of hypsarrhythmia or its variants, it should be repeated in 1-2 weeks, and once the diagnosis of IS established, the evaluations shift to classification and determination of the underlying etiology. When the history is being taken, special attention should be paid to prenatal issues and prior development. Examination may reveal dysmorphic features, neurologic signs, or neurocutaneous stigmata.

Neuroimaging is the most important diagnostic test that leads to confirmation of etiology in approximately 70% of cases. MRI is the initial neuroimaging modality of choice, with higher sensitivity in detecting subtle structural abnormality compared to CT. PET technique should be considered in medically intractable cases when focal EEG or clinical examination raise suspicion for a focal CNS process that may be amenable to surgical resection (4).

If neuroimaging or clinical examination raises suspicion of a genetic disorder, then targeted genetic testing may be indicated. Result of metabolic and genetic testing combined may determine the etiology in an additional 10% of cases. However, if the examination and neuroimaging are not revealing, a basic metabolic screen, including electrolytes, glucose, pyrovate lactate, ammonia, plasma amino acid, and urine organic acid is recommended (4).

**Fig 1:**
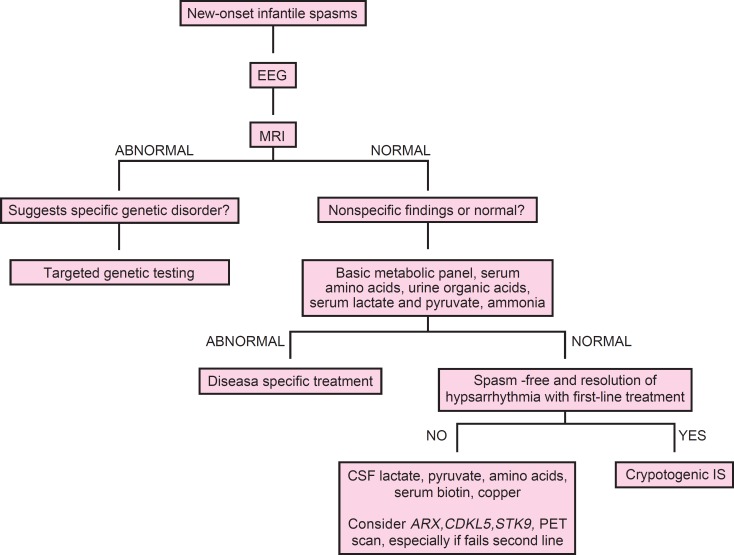
Suggested clinical evaluation and work-up of infantile spasms IS, infantile spasms; PET, positron emission tomography (4)

Genetic testing may be helpful in refractory cases without a known cause (4) (Figure1). Some other conditions may resemble IS, but they do not have the same prognosis or EEG abnormalities (1,7).

**Prognosis**

One-third of the patients died before 3 years of age and 50% before 10 years of age. Visual and auditory defects are present in one-third to one-half of affected children. Mental retardation was observed in 71 to 90 percent of patients (1). There is growing evidence that longer duration of spasms is associated with less neurodevelopmental outcomes(8). Currently, there is no evidence that medical or surgical treatment of IS can significantly alter long-term outcome (9).

A psychiatric disorder was diagnosed in 28% of patients and IS may play a significant role in the etiology of autism. Epilepsy will be seen in other forms of seizure other than IS in approximately 50% to 60% of children over the coming years. A focal lesion is often associated with a poor neurodevelopmental outcome (1). 

Spontaneous remission of spasms and disappearance of hypsarrhythimia in untreated patients have been reported in 25% of children by 1 year of age, but overall, the developmental outcome in IS is poor (4).

**Pathophysiology**

Several hypotheses have been suggested, including brainstem dysfunction of serotonergic neurons or abnormal interaction between the brainstem and a focal or diffuse cortical abnormality, abnormalities in cortical subcortical interactions. Immunologic dysfunction in the hypothalamic-adrenal-pituitary (HPA) axis has also been investigated. One hypothesis proposes that stress from variable causes in early development leads to the release of corticotropin-releasing hormone (CRH), which then causes increased neuronal excitability and seizures. Supporting evidence for this CRH excess hypothesis are decreased levels of ACTH in the cerebrospinal fluid of patients with ISs and the known efficiency of ACTH and glucocorticoids in the treatment of IS (4). 

**Treatment**

ISs are resistant to most of the conventional antiepileptic drugs. Only ACTH, corticosteroids, and vigabatrin have conclusively demonstrated effectiveness. . The most commonly used drug is ACTH in doses of 20 to 40 IU/day (1). ). Low-dose ACTH may be as effective as high-dose ACTH. In comparison with other agents, ACTH is suggested to be more effective than oral corticosteroids and compared to vigabatrin, it has improved outcome in the cessation of the spasms. For decreasing serious adverse events, such as intracranial hemorrhage, brain atrophy, Cushing syndrome, infection, weight gain and hypertension, short-term low dose therapy is recommended (10-12). In one study by Fukui et al., it has been shown that ACTH therapy can induce partial seizures for the following reasons: (1) seizure appeared only during ACTH therapy; (2) no new epileptic focus was shown by EEG, MRI, or SPECT; and (3) seizures were different from the epileptic spasms (13).

Corticosteroids are also effective; Prednisolone, hydrocortisone, and dexamethazone are most commonly recommended. Side-effects, such as hypertension, brain shrinking, cardiac hypertrophy, and adrenocortical hyporesponsiveness were related to the dose and these were therefore minimized by the drug regimen. Although synthetic ACTH (tetracosactrin) seems to produce more serious side-effects than natural corticotropin, and most authors have reported that it can be used regularly without major problems (1).

Vigabatrin has been used in the treatment of IS in Europe since 1990, and seems highly effective in spasms due to tuberous sclerosis. Benzodiazepines, most notably Nitrazepam may be effective in bringing the spasms under control, with relatively few side effects. Most investigators consider the benzodiazepine to be less active than steroids (14).

Other drugs, including Valproic acid, Zonisamide, Felbamate, Lamotrigine, Topiramate, and ketogenic diet (KD) may provide more benefit in reducing spasms and, therefore, are often used after hormonal therapy and vigabatrin (4). In a randomized controlled trial of Flunarizine as add-on therapy and effect on cognitive outcome in IS, failed to show a protective effect (15). 

KD is an effective treatment for refractory epilepsy including ISs. In one study, 17 patients were treated by KD for IS. After one month of treatment with KD, 35% of patients were seizure free, while 65% were seizure free after the third month. An early use (before 1-yearold) of KD and the use of KD after vigabatrin and steroid can be effective, and Felbamate causes an increase in the responder rate after the use of KD (16,17).

In a randomized controlled clinical trial, 60 patients with newly diagnosed and previously untreated IS, received 0.5-1 mg/kg Nitrazepam in three daily doses or 40 IU depot ACTH as a single morning dose. Following the treatments, at the end of the 6-week therapy, optimal response was obtained in 63% of patients of Nitrazepam group and 30% of patients of ACTH group. This finding supports the belief that Nitrazepam is an effective and possibly safer therapy than ACTH (18). In a descriptive study done on 60 infants aged 2-24 months in the Pediatric Neurology Department of Mofid Children’s Hospital during two years, 80% of patients were symptomatic and 20% were cryptogenic, based on clinical manifestations. 

Fifty-eight percent were flexor type, 10% extensor, and 32% mixed type. In all patients’ EEG, hypsarrhythmia was seen. Brain CT scan in 11 cases showed brain atrophy and in other cases was normal (19). in other descriptive study done on 40 cases of tuberous sclerosis in the Pediatric Neurology Department of Mofid Children’s Hospital, the most common clinical manifestation was seizure, and the most common type of seizure was myoclonic. In 19 cases with tuberous sclerosis, in the first year of life, seizure was appeared as ISs (20). The optimum evaluation and treatment of children with ISs is unknown. To aid in the development of a standardized approach for ISs, members of the Child Neurology Society were surveyed to determine common practice. The survey had 222 responders with a responder rate of 18.5%. It is important that the diagnostic evaluation and the use of first-line treatments varied among the responders. Thus, future clinical trials will require multicenter collaboration. An important first step in such collaboration is to standardize the evaluation and treatment of IS within and between participating centers (21, 22).
